# Association between (GT)n Repeats in Heme Oxygenase-1 Gene Promoter and 3-Year Survival of Patients with Acute Leukemia: a Controlled, Cross-Sectional Study

**Published:** 2018-01-01

**Authors:** Mohammad Kazemi, Farinaz Khosravian, Amir Abbas Sameti, Alireza Moafi, Mohammad Reza Merasi, Mansour Salehi, Majid Nejati, Mohaddeseh Behjati

**Affiliations:** 1 Department of Genetics and Molecular Biology, School of Medicine, Isfahan University of Medical Sciences, Isfahan, Iran; 2 Pediatric Inherited Diseases Research Center, Research Institute for Primordial Prevention of Non-Communicable Disease, Isfahan University of Medical Sciences, Isfahan, Iran; 3 Genome Medical Genetics Center, Isfahan University of Medical Sciences, Isfahan, Iran; 4 Isfahan Dental Student Research Committee, School of Dentistry, Isfahan University of Medical Sciences, Isfahan, Iran; 5 Department of Pediatric Hematology, School of Medicine, and Child Health Promotion Research Center, Isfahan University of Medical Sciences, Isfahan, Iran; 6 Department of Epidemiology and Biostatistics, School of Health, Isfahan University of Medical Sciences, Isfahan, Iran; 7 Anatomical Sciences Research Center, Kashan University of Medical Sciences, Kashan, Iran; 8 Rajaie Cardiovascular Medical and Research Center, Iran University of Medical Sciences, Tehran, Iran

**Keywords:** Acute leukemia, Survival, GT repeats, Heme-oxygenase-1 gene promoter

## Abstract

**Background:** Acute leukemia is a common pediatric cancer. Novel strategies for treatment of acute leukemia have been developed, but treatment resistance is remained as the most problematic issue. It is hypothesized that the *HO-1 *gene up-regulation is responsible for tumor resistance to chemotherapy or radiotherapy-induced apoptosis. The levels of *HO-1* expression are related to (GT)_n_ microsatellite polymorphisms in the location of its promoter. This study designed to compare allelic frequencies of (GT)_n_ microsatellite polymorphisms in *HO-1* gene between acute leukemia patients and healthy controls. Indeed, 3-year disease-free survival was also evaluated.

**Methods:** Sixty-three patients with acute leukemia and seventy healthy infants were included in this study. We used the medical records of patients to collect information about survival after chemotherapy. The number of GT repeats in *HO-1* promoter was determined by an ABI 3100 sequencer.

**Results:** The *HO-1* GT repeats ranged from 14 to 34 with peaks at 27 repeats in both cases and controls. Children with longer alleles ((GT)_n _≥ 27) had enhanced 3-year survival rate after treatment with chemotherapy or radiotherapy (P<0.05).

**Conclusion:** Although no significant differences were observed between leukemia patients and controls regarding allelic frequency, we found elevated frequency of “LL” genotype in leukemia patients with good prognosis and 3-year surveillance. Radiotherapy and chemotherapy might elevate the expression levels of *HO-1* with subsequent increased resistance of leukemia patients to therapy.

## Introduction

 Acute Leukemia is the most common pediatric cancer ^1^^,^^2^ . Despite numerous efforts focused on leukemia treatment, this type of disease remains one of the greatest challenges in pediatric oncology^   3 ^. Current therapeutic strategies are unable to provide long-term remission for 20% of children with ALL (25-40% cure rate after relapse)^   4 ^. Some subgroups of infants are likely to be over treated and might be healed using less intensive regimens which results in reduced toxicity and fewer long-term side effects ^5^^-^^7^ . Response to treatments is variable depending on different clinical, immunological, cytogenetic/genetic characteristics and environmental exposures, which are associated with increased risk of leukemia and their diverse response to treatment ^8^^-^^12^^.^

One of the mentioned genetic factors is Heme Oxygenase-1 gene (*HO-1*), located on chromosome 22. This gene produces an essential enzyme in heme catabolism. It has 2 isozymes, an inducible heme oxygenase-1 and a constitutive heme oxygenase-2^   13 ^. *HMOX1* and *HMOX2* belong to the heme oxygenase family. Heme Oxygenase (*HO*) gene encodes an enzyme which catalyzes the first and rate-limiting steps in oxidative degradation of heme to the form of open-chain tetrapyrrole biliverdin-IX with final release of carbon dioxide, biliverdin and free iron . By now, three isozymes of mammalian *HO* have been identified, in which *HO-1* variant is the only inducible isozyme with expression in different normal and neoplastic cells. *HO-1* expression in malignant tissues is higher than its surrounding healthy tissues which could be up regulated by different stimuli and oxidative stresses such as radiotherapy, chemotherapy and photodynamic therapies ^14^^-^^20^ . 

*HO-1* is involved in pathogenesis of acute leukemia patients and their resistance to treatment ^8^^-^^11^ . Expression of HO-1 has the protective and anti-apoptotic effects through its active biological products*.* ^21^^,^^22^ . As a result, it's possible that the upregulation of HO-1 is responsible for increasing tumor resistance to chemotherapy or radiotherapy. ^23^^, ^^24^ . However, *HO-1* expression level is dependent to its polymorphisms and fluctuates between individuals, quantitatively ^25^^, ^^26^ .

GT repeats in *HO-1* gene promoter modulate transcription of *HO-1* gene. The dinucleotide sequence can adversely affect the basal promoter activity ^27^^-^^30^ . GT repeats ranged between 12 - 40 base pairs. This range can be divided into short “S” and long “L” alleles although there is not an exact cut-off to separate two alleles^16^^,^^31^^,^^32^. Moreover, some surveys revealed higher prevalence of "S" allele in renal cell carcinoma, pancreatic cancer, gastric cancer and gastric adenocarcinoma^   33 ^. Paradoxically, patients with squamous cell carcinoma and lung adenocarcinoma often carry “L” allele^16^^,^^31^^,^^34^^-^^37^. Some studies revealed that *HO-1 *targeting with either pegylated zinc protoporphyrin (PEG-ZnPP) or styrene maleicacid-micelle–encapsulated ZnPP (SMAZnPP) results in growth inhibition in cancer cells with subsequent increased sensitivity to radiotherapy and chemotherapy ^22^^,^^38^^-^^40^ . 

Despite advent of novel strategies for treatment of acute leukemia, resistance to treatment is the greatest concern until now. *HO-1* over expression provides cytoprotective and antiapoptotic effects on malignant cells against radiotherapy and chemotherapy. Although the role of *HO-1* in AML and CML cell lines has been studied, the distribution of allele frequency in leukemia and its association with resistance to treatment and survival remains unveiled^   10 ^. This study was designed to compare the allelic frequencies of genetic variants in the promoter of HO-1 among patients suffering from acute leukemia compared with healthy controls. The relationship between GT repeats and patient’s 3-year and 5-year survival was also assessed. 

## MATERIALS AND METHODS

**Patient selection**

This is a controlled, cross-sectional study that included 63 patients with acute leukemia admitted to Seyed-al-Shohada Hospital, Isfahan from 2006 to 2009. The control group consisted of 54 gender and age-matched healthy children. Our data about survival after chemotherapy and bone graft were gathered from patients’ medical records. DNA was extracted from bone marrow samples of the patients. Genomic DNA was extracted through QIAamp DNA blood mini kit (QIAGEN, USA) according to the manufacturer’s instruction. The study protocol was approved by the Ethics Board of Isfahan University of Medical Sciences

**Heme oxygenase-1 genotyping**

The *HO-1* microsatellite was amplified through PCR using a 5'-FAM-labeled forward primer (AGAGCCTGCAGCTTCTCAGA) and a 3'-unlabelled reverse primer (GTCCTATGGCCAGACTTTGT), in 30 cycles (94°C for 30 seconds, 60°C for 30 seconds, and 72°C for 30 seconds) and final extension at 72°C for 5 minutes by thermo cycler (Eppendorf Mastercycler EP Gradient, USA). Labeled PCR products were compared with a standard size marker GenoType^TM^ TAMRA DNA ladder (size range 50–500 bp) (Gibco-BRL, Paisley, Scotland, UK). Number of GT repeats was determined with an ABI 3100 sequencer (Applied Biosystems, USA), using GeneMarker V1.97 software (Softgenetics, USA). Selected samples were sequenced by ABI 3100 sequencer (Applied Biosystems, USA). 

**Statistical** **analysis**

Statistical analyses were performed using SPSS 16.0. Chi-square test was used for categorical variables. Three-year survival was compared with allelic frequencies of *HO-1* polymorphism. Differences in alleli c frequency of *HO-1* promoter were compared between leukemia patients and controls. Difference of data with *P*< 0.05 was considered as a significant. 

## Results

 Demographic characteristics of patients including age and sex are shown in Table 1. Frequency distribution of age and sex was not significantly different between studied groups. Patients whose data were incomplete were excluded from the study.

**Table1:** Demographic characteristics of patients (age and sex)

**Group**			**Case**	**control**	**P-value**
Age (years) Mean ±SD			5.93 ±0.63	4.87±0.26	>0.05
Gender (%)	F		59.5	46.2	>0.05
	M		40.5	53.8	

**Figure 1:**
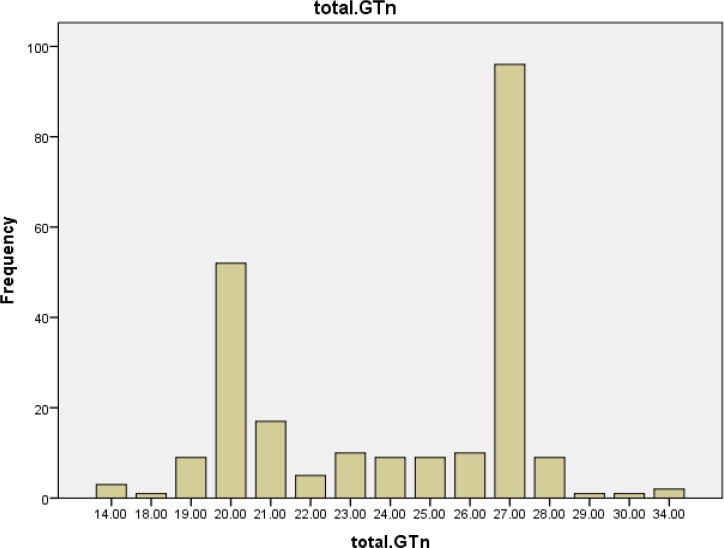
Frequency distributions of (GT) repeats in all participants (n=117)

**Figure 2:**
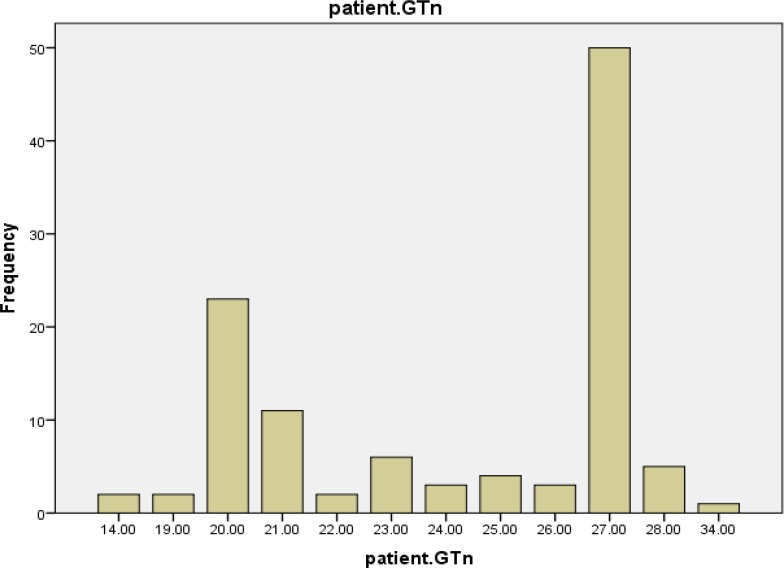
Frequency distributions of (GT) repeats in leukemia patient (n=56)

**Figure 3:**
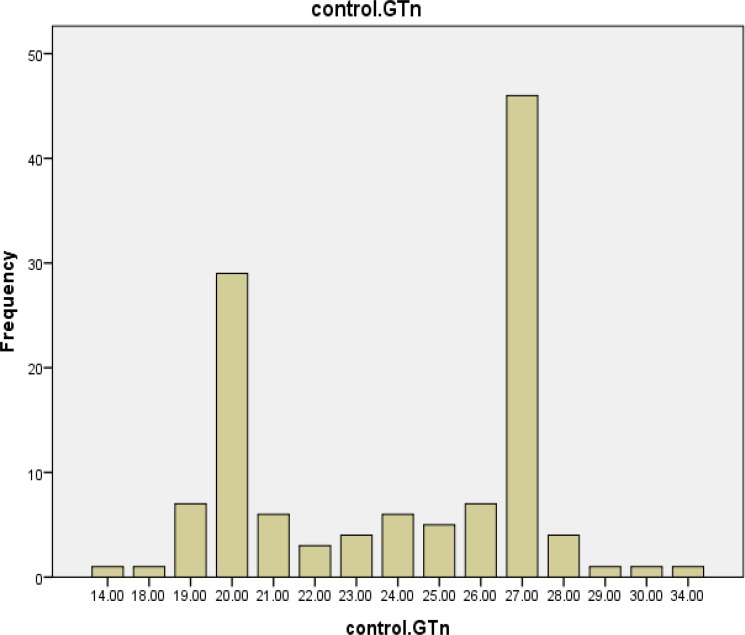
Frequency distributions of (GT) repeats in control group (n=61)

The *HO-1* genotyping process for 63 cases and 54 healthy controls was performed successfully. The alleles frequency of (GT)_n_ microsatellite polymorphism of HO -1 gene promoter was found in both case and control groups (Figures 1, 2 and 3). The GT repeat numbers ranged from 14 to 34, and the most prevalent allele in our population was (GT)_27_. Therefore, we divided these alleles into two subgroups. (GT)_n_ : short (S) alleles <27 repeats and long (L) alleles ≥27 repeats. Three genotypes (SS, SL and LL) were created for the study population. The subjects with genotype SS and SL were placed in a group identified as "short carrier genotype" and those with LL genotype were placed in a group identified as "long genotype".

There was no significant difference in the frequency of genotypes between leukemia patients and controls (P= 0.629) (Table 2). The "long genotype" was more frequent in leukemia patients with better vs. worse 3-year surveillance (*P*< 0.05)(Table 3). Furthermore, there was no significant difference in hepatomegaly, splenomegaly and CSF metastasis in two genotype groups (P≥0.05) (Table 4). 

**Table 2:** Genotype* distribution in case and control groups

	**Genotypes**			**P-value**
	LL	LS	SS	0.629
Case	33.3%	47.6%	19%	
Control	33.3%	40.7%	25.9%	

* (GT)n < 27 Repeats: S allele, (GT)n ≥ 27 Repeats: L allele, SS: Homozygote for Short Allele, LL: Homozygote for Long Allele, LS: Heterozygote Person

**Table 3:** Genotype* distribution for leukemia patients according to 3-and 5-year survival, respectively

		**Genotype**		**P-value**
		S carrier	LL	
3-year survival rate				
	Yes	38.8%	30.5%	0.043
	No	27.7%	2.7%	
5-year survival rate				
	Yes	39.3%	21.2%	0.371
	No	30.3%	9.0%	

(GT)n < 27 Repeats: S allele, (GT)n ≥ 27 Repeats: L allele, SS: Homozygote for Short Allele, LL: Homozygote for Long Allele, LS: Heterozygote Person

**Table 4:** Genotype* distribution of leukemic regarding hepatomegaly, splenomegaly and CSF metastasis

		**Genotypes **		**P-value**
		S carrier	LL	
Hepatomegaly	(+) (-)	21.0% 50.0%	15.85 13.1%	0.142
Splenomegaly	(+) (-)	40.5% 29.7%	16.2% 13.5%	0.571
CSF Metastasis	(+) (-)	2.9% 67.6%	0.0% 29.4%	0.706

* S carrier: a person who is carrier for S allele, LL: Homozygote for L allele, CSF Metastasis: Metastasis to Cerebrospinal fluids (CSF)

## Discussion

 In this study, we investigated polymorphic (GT)_n_ repeats in *HO-1* promoter region in patients suffering from leukemia. In addition, we investigated the association between allelic frequency and patients’ 3-year surveillance. We found no relationship between frequency distribution of (GT)_n_ repeats and leukemia incidence. We have observed that patients carrying “LL” allele had significantly better 3-year survival. Five-year survival was slightly better in carriers of “LL” allele although this difference was not statistically significant. Moreover, we studied genotype distribution among patients considering hepatomegaly, splenomegaly and CSF metastasis (Table 4), which revealed no relationship between (GT)n repeats in *HO-1* promoter and clinical features of patients. 

HO-1 gene is induced in response to oxidative stress such as chemotherapy and radiotherapy^41^. The basal and induced levels of HO-1 gene expression are different in each individual. The polymorphism length of *HO-1,* influences the level of gene transcription. Excessive GT repeats reduced promoter activity, while short GT alleles led to higher levels of *HO-1* expression with further anti-apoptotic effects. Our results suggest that “SS” and “SL” genotypes make tumors more resistant to anticancer therapy in acute leukemia. 

*HO-1* considered as a “friend” which supports normal tissues against carcinogen-induced invasion ^42^^,^^43^ Nevertheless, during the neoplastic development, *HO-1* turns into a “false friend” and facilitate tumor development. *HO-1* over expression in CML tumor cells increases its viability through apoptosis inhibition.

Meyerhof et al. have treated CML cells by zinc-(II)-deuteroporphyrin-IX, inhibitor of HO-1 and hemin, inducer of an HO-1. Parallel usage of HO-1 inhibitors and inducers, decreases and increases CML cells viability in stress oxidative environment, respectively. Also, it has been apparent that HO-1 reduces sensitivity of cancer cells to chemotherapy and radiotherapy^22^^,^^44^. 

The length polymorphism of GT repeats in *HO-1* gene promoter had various impacts in different disease states. Chang et al*.* reported an increased risk of oral squamous cell carcinoma in patients with more than 31 GT-repeats^        45 ^. Moreover, some surveys revealed a higher frequency of "S" allele in renal cell carcinoma, pancreatic cancer, gastric cancer, gastric adenocarcinoma, lymphoma and Kaposi sarcoma. In this study, the frequency of “S” alleles and “L” alleles were equal in both leukemia patients and control groups.

This study is the first to investigate *HO-1* gene promoter polymorphism and surveillance (3 years) in leukemia patients. Although we found no significant differences between leukemia patients and control groups regarding short (GT) n repeat alleles in *HO-1* promoter, higher frequency of “LL” genotype in leukemia patients with positive 3-year surveillance was found. Indeed, LL genotype in leukemia patients was associated with slightly better 5-year surveillance. 

Oxidative stress conditions such as radiotherapy and chemotherapy lead to additional expression of *HO-1* gene^        41 ^. These data supports the idea that higher levels of *HO-1* gene expression, associated with “SS” and “SL” genotype, might play an important role in elevated resistance of leukemia patients to chemotherapy or radiotherapy.

## CONCLUSION

 Modification of *HO-1* expression level might have positive effect on the prognosis of leukemia patients under treatment. Further studies are required to determine the impact of *HO-1* genotype in patients with acute leukemia.

## References

[1] Lo Nigro L (2013). Biology of childhood acute lymphoblastic leukemia. J Pediatr Hematol Oncol..

[2] Arora RS, Arora B (2016). Acute leukemia in children: A review of the current Indian data. South Asian J Cancer..

[3] Hunger SP, Raetz EA, Loh ML (2011). Improving outcomes for high‐risk ALL: Translating new discoveries into clinical care. Pediatr Blood Cancer..

[4] Conter V, Arico M, Basso G (2010). Long-term results of the Italian Association of Pediatric Hematology and Oncology (AIEOP) Studies 82, 87, 88, 91 and 95 for childhood acute lymphoblastic leukemia. Leukemia..

[5] Hunger SP, Winick NJ, Sather HN (2005). Therapy of low-risk subsets of childhood acute lymphoblastic leukemia: when do we say enough?. Pediatr Blood Cancer..

[6] Arico M, Conter V, Valsecchi MG (2005). Treatment reduction in highly selected standard-risk childhood acute lymphoblastic leukemia. The AIEOP ALL-9501 study. Haematologica..

[7] Van Dongen-Melman JE, De Groot A, Van Dongen JJ (1997). Cranial irradiation is the major cause of learning problems in children treated for leukemia and lymphoma: a comparative study. Leukemia..

[8] Dorantes-Acosta E, Pelayo R (2012). Lineage Switching in Acute Leukemias: A Consequence of Stem Cell Plasticity?. Bone Marrow Res..

[9] Belson M, Kingsley B, Holmes A (2007). Risk factors for acute leukemia in children: a review. Environ Health Perspect..

[10] Heasman SA, Zaitseva L, Bowles KM (2011). Protection of acute myeloid leukaemia cells from apoptosis induced by front-line chemotherapeutics is mediated by haem oxygenase-1. Oncotarget..

[11] Siegel R, Naishadham D, Jemal A (2012). Cancer statistics, 2012. CA Cancer J Clin..

[12] Bassan R, Hoelzer D (2011). Modern therapy of acute lymphoblastic leukemia. J Clin Oncol..

[13] Ryter SW, Alam J, Choi AM (2006). Heme oxygenase-1/carbon monoxide: from basic science to therapeutic applications. Physiol Rev..

[14] Kiemer AK, Bildner N, Weber NC (2003). Characterization of heme oxygenase 1 (heat shock protein 32) induction by atrial natriuretic peptide in human endothelial cells. Endocrinology..

[15] Immenschuh S, Ramadori G (2000). Gene regulation of heme oxygenase-1 as a therapeutic target. Biochem Pharmacol..

[16] Exner M, Minar E, Wagner O (2004). The role of heme oxygenase-1 promoter polymorphisms in human disease. Free Radic Biol Med..

[17] Salinas M, Diaz R, Abraham NG (2003). Nerve growth factor protects against 6-hydroxydopamine-induced oxidative stress by increasing expression of heme oxygenase-1 in a phosphatidylinositol 3-kinase-dependent manner. J Biol Chem..

[18] Martin D, Rojo AI, Salinas M (2004). Regulation of heme oxygenase-1 expression through the phosphatidylinositol 3-kinase/Akt pathway and the Nrf2 transcription factor in response to the antioxidant phytochemical carnosol. J Biol Chem..

[19] Malaguarnera L, Imbesi RM, Scuto A (2004). Prolactin increases HO-1 expression and induces VEGF production in human macrophages. J Cell Biochem..

[20] Lam CW, Getting SJ, Perretti M (2005). In vitro and in vivo induction of heme oxygenase 1 in mouse macrophages following melanocortin receptor activation. J Immunol..

[21] Fang J, Akaike T, Maeda H (2004). Antiapoptotic role of heme oxygenase (HO) and the potential of HO as a target in anticancer treatment. Apoptosis..

[22] Mayerhofer M, Florian S, Krauth MT (2004). Identification of heme oxygenase-1 as a novel BCR/ABL-dependent survival factor in chronic myeloid leukemia. Cancer Res..

[23] Jozkowicz A, Was H, Dulak J (2007). Heme oxygenase-1 in tumors: is it a false friend?. Antioxid Redox signal..

[24] Yajima T, Ochiai H, Uchiyama T (2009). Resistance to cytotoxic chemotherapy-induced apoptosis in side population cells of human oral squamous cell carcinoma cell line Ho-1-N-1. Int J Oncol..

[25] Tang D, Tang W-J, Shi X-L (2016). Association of the microsatellite (GT) n repeat polymorphisms of the HO-1 gene promoter and corresponding serum levels with the risk of laryngeal squamous cell carcinoma. Acta Oto-Laryngologica..

[26] Køllgaard T, Kornblit B, Petersen J (2016). (GT)n Repeat Polymorphism in Heme Oxygenase-1 (HO-1) Correlates with Clinical Outcome after Myeloablative or Nonmyeloablative Allogeneic Hematopoietic Cell Transplantation. PloS one..

[27] Rich A, Nordheim A, Wang AH (1984). The chemistry and biology of left-handed Z-DNA. Annu Rev Biochem..

[28] Naylor LH, Clark EM (1990). d(TG)n.d(CA)n sequences upstream of the rat prolactin gene form Z-DNA and inhibit gene transcription. Nucleic Acids Res..

[29] Delic J, Onclercq R, Moisan-Coppey M (1991). Inhibition and enhancement of eukaryotic gene expression by potential non-B DNA sequences. Biochem Biophys Res Commun..

[30] Waring GO 3rd, Laibson PR (1977). Keratoplasty in infants and children. Trans Sect Ophthalmol Am Acad Ophthalmol Otolaryngol.

[31] Sawa T, Mounawar M, Tatemichi M (2008). Increased risk of gastric cancer in Japanese subjects is associated with microsatellite polymorphisms in the heme oxygenase-1 and the inducible nitric oxide synthase gene promoters. Cancer Lett..

[32] Javanmard SH, Keyhanian K, Loghmani P (2012). Association between heme oxygenase-1 gene promoter polymorphisms and metabolic syndrome in Iranians. Mol Biol Rep..

[33] Lo SS, Lin SC, Wu CW (2007). Heme oxygenase-1 gene promoter polymorphism is associated with risk of gastric adenocarcinoma and lymphovascular tumor invasion. Ann Surg Oncol..

[34] Berberat PO, Dambrauskas Z, Gulbinas A (2005). Inhibition of heme oxygenase-1 increases responsiveness of pancreatic cancer cells to anticancer treatment. Clin Cancer Res..

[35] Marinissen MJ, Tanos T, Bolós M (2006). Inhibition of heme oxygenase-1 interferes with the transforming activity of the Kaposi sarcoma herpesvirus-encoded G protein-coupled receptor. J Biol Chem..

[36] Sunamura M, Duda DG, Ghattas MH (2003). Heme oxygenase-1 accelerates tumor angiogenesis of human pancreatic cancer. Angiogenesis..

[37] Yanagawa T, Omura K, Harada H (2004). Heme oxygenase-1 expression predicts cervical lymph node metastasis of tongue squamous cell carcinomas. Oral Oncol..

[38] Mayerhofer M, Gleixner KV, Mayerhofer J (2008). Targeting of heat shock protein 32 (Hsp32)/heme oxygenase-1 (HO-1) in leukemic cells in chronic myeloid leukemia: a novel approach to overcome resistance against imatinib. Blood..

[39] Fang J, Sawa T, Akaike T (2004). Enhancement of chemotherapeutic response of tumor cells by a heme oxygenase inhibitor, pegylated zinc protoporphyrin. Int J Cancer..

[40] Nowis D, Legat M, Grzela T (2006). Heme oxygenase-1 protects tumor cells against photodynamic therapy-mediated cytotoxicity. Oncogene..

[41] Was H, Sokolowska M, Sierpniowska A (2011). Effects of heme oxygenase-1 on induction and development of chemically induced squamous cell carcinoma in mice. Free Radic Biol Med..

[42] Ferrando M, Wan X, Meiss R (2013). Heme oxygenase-1 (HO-1) expression in prostate cancer cells modulates the oxidative response in bone cells. PloS one..

[43] Labanca E, De Luca P, Gueron G (2015). Association of HO-1 and BRCA1 is critical for the maintenance of cellular homeostasis in prostate cancer. Mol Cancer Res..

[44] Traverso N, Ricciarelli R, Nitti M (2013). Role of glutathione in cancer progression and chemoresistance. Oxid Med Cell Longev.

[45] Chang KW, Lee TC, Yeh WI (2004). Polymorphism in heme oxygenase-1 (HO-1) promoter is related to the risk of oral squamous cell carcinoma occurring on male areca chewers. Br J Cancer..

